# Synthesis of New Monoazo Disperse Dyes for Dyeing Polyester Fabric Using Two Different Dyeing Methods: Demonstration of Their Antibacterial and Anticancer Activities

**DOI:** 10.3390/polym15143052

**Published:** 2023-07-15

**Authors:** Khaled M. A. Abdelmoteleb, Morsy A. El-Apasery, Ashraf A. F. Wasfy, Sara M. Ahmed

**Affiliations:** 1 Chemistry Department, Faculty of Science, Benha University, Benha 13518, Egypt; khaled.mrm79@gmail.com (K.M.A.A.); ashrafwasfy@rocketmail.com (A.A.F.W.); 2 Dyeing, Printing and Textile Auxiliaries Department, Textile Research and Technology Institute, National Research Centre, 33 El Buhouth St., Dokki, Cairo 12622, Egypt; 3 Applied Biosciences and Process Engineering Department 7, Analt University of Applied Sciences, Bernburger Street 55, P.O. Box 1458, 06366 Köthen, Germany; selapasery@gmail.com

**Keywords:** disperse dyes, polyester fabrics, fastness properties, antibacterial, anticancer

## Abstract

3-(dimethylamino)-1-phenylprop-2-en-1-ones were obtained with good yields by reacting dimethylformamide dimethylacetal with different methyl ketones. 3-oxo-3-phenyl-2-(2-phenylhydrazono)propanals disperse dyes were obtained via reacting of 3-(dimethylamino)-1-phenylprop-2-en-1-ones with phenyldiazonium chloride. The novel dyes were used in dyeing polyester fabrics through two different dyeing methods at temperatures of 100 and 130 °C. We found that the color strength when using the dyeing method at 130 °C was better than the dyeing method at 100 °C. The fastness properties of dyed fabrics with these new disperse dyes were studied and gave very good results (except for fastness to light, which gave moderate results). The new dyes were evaluated against some different types of bacteria and cancer, which showed excellent and promising results for the possibility of using these dyes as antibacterial and anticancer agents.

## 1. Introduction

Due to its great strength and thermal stability, polyester is employed in a wide range of textile applications (largely due to is capability with natural fibres). However, due to its high crystallinity, compactness, water repellency, and lack of chemically reactive groups, polyester-based textiles have a number of disadvantages, including low moisture content, pollution, a bad feeling, and difficulty in dyeing. As a result of this, significant efforts have been made to raise the quality of the dye by upgrading techniques such as carrier-free dyeing, alkaline media dyeing, and hot and high pressure dyeing [1,2,3,4,5,6].

For polyester and other hydrophobic fabrics, disperse dye is a form of synthetic dye [7,8,9]. Being the most hydrophobic fibre among common fibres, polyester takes a lot of energy during water dyeing in order to adsorb the disperse dyes. These fibres are affordable raw materials that are simple to get and have desirable qualities including high strength, light weight, and great dyeability. Polyester fibre is extremely hydrophobic and has a crystalline, compact structure. As a result, its aqueous dyeing is carried out using disperse dyes at low or high temperatures.

Dissolving and resolving disperse dyes, transporting disperse dyes from bulk solutions to the fibre surface, diffusion and adsorption of dye at the surface, and diffusion from the surface into the fiber’s interior are all steps in the polyester dyeing process. In order to reduce effluent loading and enhance environmental friendliness of textile production, effective fundamental adjustments to the dyeing processes are necessary. One of these changes is the use of modern techniques that make the dyeing process affordable, quick, and labor-intensive while also being sustainable. In order to increase color yield, reduce the amount of wastewater generated during the textile manufacturing process, and enhance fabric absorption, sustainable energy sources such gamma radiation, ultrasound, infrared radiation, plasma, and microwaves are used. In order to colour polyester materials while saving time and energy, microwave radiation or ultrasound has been used as a crucial component. In order to be quicker and more effective than the traditional heating method, microwave heating has evolved. In this instance, microwave energy is an efficient approach to lessen heat transfer issues since it can immediately heat up all of the particles inside the molecule. The suitable dyeing time is very important to disintegrate and redisintegrate dye dispersion, polyester structure opening and swelling, and soluble dye transfer from the dye liquid to the surface of the fiber. The dye is adsorbed on the surface of the fiber, and then the dye is molecularly diffused into the fiber structure. Temperature is crucial when using disperse dye since it affects the dyeing process. If a high temperature dyeing process is used, temperature exceeding 100 °C is necessary to prevent fibres from expanding. Similar to the carrier coloring process, this swelling happens between 85 and 90 degrees. A longer storage period could result in dye sublimation and a loss of fabric tenacity. An organic substance called a carrier quickens the dyeing process by breaking down or dissolving colour aggregates and transporting them in tiny enough quantities to the fiber-water interface to be absorbed by the fabric. Here, we can discuss the advantages of dying with a carrier since it is challenging to dye highly crystalline polyester materials in a deep shade when using the conventional dyeing procedure, which is carried out at temperatures lower than 100 °C. At 100 degrees Fahrenheit, a carrier can produce a medium to dark hue. Additionally, dyeing fabrics is a simple process that may be carried out at atmospheric pressure and below 100 degrees Celsius. Thus, polyester fabric can be dyed at a medium level. One of the key advantages of carrier materials is that it lessens the staining of wool during the dyeing of blends of wool and polyester, enhances the fabric’s fastness characteristics, such as fastness to washing, perspiration, and friction, speeds up dyeing, and raises the dyeing rate. It is important to note that dyeing with a carrier has significant issues such as an unpleasant odour, toxicity, and environmental contamination. Thus we had to utilise a carrier that was safe for the environment [10,11,12,13,14,15,16,17,18,19,20,21,22,23,24,25].

Disperse dyes are polar molecules having azo groups. An estimated 43% of disperse dyes are made with azos dyes. Because they are so simple to make, 3-(dimethylamino)-1-phenylpropenones (enaminones) have garnered a lot of attention in chemistry [10]. Numerous 3-(dimethylamino)-1-phenylpropenone derivative-based dispersion dyes [12,13,14,15,16,17,18] have been created. A valuable intermediate in fine chemical synthesis, 3-(dimethylamino)-1-phenylpropenones are created when a primary or secondary amine interacts with a diketone in an aromatic solvent. This type of disperse azo dye, which is based on 3-(dimethylamino)-1-phenylpropenones, has vivid hues [2,5].

Derivatives of 3-(dimethylamino)-1-phenylpropenones-based compounds have been used [19,20,21,22] as a precursor for the incorporation of heterocyclic rings with a range of biological activities. The goal of this effort was to safely and easily synthesize new disperse azo dyes based on derivatives of 3-(dimethylamino)-1-phenylpropenones. This study looked at the performance of disperse dyes **7a–f** when a dyeing procedure was used at 100 or 130 °C. The dyeing performance employing the dyed polyester fabrics’ color strength was presented using the K/S estimation. Finally, we investigated if these new disperse dyes have antibacterial and anticancer capabilities as an added benefit to these new disperse dyes. Fastness characteristics of dyed polyester fabrics were also evaluated.

## 2. Materials

The NMR spectra were analyzed at 300 MHz using a Mercury-300BB spectrometer. Comparing substance changes to a tetramethylsilane internal standard, ppm values were recorded. The Fourier-change infrared (FT-IR) spectra were controlled using a JASCO FT/IR4700 spectrophotometer. The elemental analyses (C, H, and N) were performed using the PerkinElmer 2400 analyzer (PerkinElmer, Norwalk, CT, United States). The solvents utilized in this exploration inquiry for both the synthesis methods and the spectroscopic estimations were given by Fluka and Aldrich.

**General procedures for preparing of 3-(dimethylamino)-1-phenylpropenones 3a–f**.

In accordance with the directions outlined in our earlier work [12,13,14,15,16,17,18], compounds **3a**–**f** were created. Acetophenone, m-methyl acetophenone, m-methoxy acetophenone, m-bromo acetophenone, and m-nitro acetophenone were used in a mixture to reflux dimethylformamide dimethylacetal (DMFDMA) (1.19 g, 0.01 mol) for 12 to 16 h. The completeness of the reactions was monitored by thin layer chromatography (TLC). After cooling to room temperature, petroleum ether was used to treat the reaction mixture. The solid byproduct that had been created was filtered out and collected in order to produce chemicals

**General procedures for synthesis of disperse dyes 7a–f.**

A cold solution of the diazonium salt **4** [(10 mmol) (prepared by adding a cold solution of sodium nitrite (0.7 g) in water (5 mL) to a solution of the aniline (10 mmol) in conc. HCl (5 mL) at a temperature 0–5 °C] was added to a cold solution of enaminone **3a–f** (10 mmol) in ethanol (10 mL) containing sodium sulfate (1 g). The mixture was stirred at temperature < 5 °C for 1 h. The solid precipitate that formed was collected by filtration and crystallized using the proper solvents to obtain a yellow-orange crystals.

***3-oxo-3-phenyl-2-(2-phenylhydrazono)propanal 7a:***

Yield 1.96 g (76%); mp 83 °C (Lit. [6] mp 82 ± 84 °C).

***3-oxo-2-(2-phenylhydrazono)-3-m-tolylpropanal 7b***

Yield (80%); Anal. Calcd For C_16_H_14_N_2_O_2_: (266.29), C, 72.16; H, 5.30; N, 10.52. Found: C, 72.40; H, 5.43; N, 10.68; MS *m*/*z* (M-1)^+^ = 265.42; IR: 3430, 3050, 2915, 1644, 1590 cm^−1^; ^1^H NMR (DMSO-*d*_6_): δ = 2.37 (t, 3H, CH_3_), 7.11–7.69 (m, 9H, arom-H), 10.00 (s, 1H, CHO), 14.20 (s, 1H, NH)

***3-(3-methoxyphenyl)-3-oxo-2-(2-phenylhydrazono)propanal 7c***

Yield (81%); Anal. Calcd For C_16_H_14_N_2_O_3_: (282.29), C, 68.07; H, 5.00; N, 9.92. Found: C, 67.98; H, 5.13; N, 10.15; MS *m*/*z* (M-1)^+^ = 281.02; IR: 3441, 3059, 2924, 1643, 1590 cm^−1^; ^1^H NMR (DMSO-*d*_6_): δ = 3.79 (t, 3H, CH_3_), 7.17–7.49 (m, 9H, arom-H), 10.00 (s, 1H, CHO), 14.23 (s, 1H, NH).

***3-(3-chlorophenyl)-3-oxo-2-(2-phenylhydrazono)propanal 7d***

Yield (72%). Anal. Calcd For C_15_H_11_ClN_2_O_2_: (286.71), C, C, 62.84; H, 3.87; N, 9.77. Found: C, 62.97; H, 4.05; N, 9.94; MS *m*/*z* (M-1)^+^ = 285.37; IR: 3432, 3035, 2919, 1644, 1590 cm^−1^; ^1^H NMR (DMSO-*d*_6_): δ = 7.15–7.88 (m, 9H, arom-H), 10.00 (s, 1H, CHO), 14.21 (s, 1H, NH).

***3-(3-bromophenyl)-3-oxo-2-(2-phenylhydrazono)propanal 7e***

Yield (73%). Anal. Calcd for C_15_H_11_BrN_2_O_2_: (331.16), C, 54.40; H, 3.35; N, 8.46. Found: C, 54.68; H, 3.51; N, 8.70; MS *m*/*z* (M)^+^ = 331.06; IR: 3429, 3030, 2920, 1644, 1591 cm^−1^; ^1^H NMR (DMSO-*d*_6_): δ = 7.18–8.03 (m, 9H, arom-H), 10.00 (s, 1H, CHO), 14.20 (s, 1H, NH).

***3-(3-nitrophenyl)-3-oxo-2-(2-phenylhydrazono)propanal 7f***

Yield (75%). Anal. Calcd for C_15_H_11_N_3_O_4_: (297.27), C, 60.61; H, 3.73; N, 14.14. Found: C, 60.89; H, 3.80; N, 14.31; MS *m*/*z* (M-2)^+^ = 295.84; IR: 3437, 3081, 1643, 1523 cm^−1^; ^1^H NMR (DMSO-*d*_6_): δ = 7.19–8.68 (m, 9H, arom-H), 10.02 (s, 1H, CHO), 14.18 (s, 1H, NH).

### 2.1. Fabric

Scoured and bleached 100% polyester fabric (149 g/m^2^) was supplied by El-Mahalla El-Kobra Company. The fabrics were scoured in aqueous solution with a liquor ratio1:30 containing 2 g/L nonionic detergent solution (Hostapal, Clariant) and 2 g/L sodium carbonates at 50 °C for 30 min to remove impurities, then rinsed thoroughly in cold tap water, and dried at room temperature.

### 2.2. Dyeing Procedure

The appropriate amount of dyes (3% shades) were dissolved in 2 mL dimethylformamide (DMF) to create a dispersion of disperse dyes **7a**–**f**, which was then added dropwise with stirring to the dye bath (liquor ration: 1:30) containing (1.5%) of levegal MDL as an anionic dispersing agent and (1%) of TANAVOL EP 2007 as an anionic eco-friendly carrier in the case of dyeing at 100 °C or without carrier in case of dyeing at130 °C. When the pH of the dye bath was adjusted to 5.5, the polyester fabrics were added. The dye bath has to be heated to 100 °C at a rate of 3 °C/min and maintained there for 60 min. After being reduced (1 g/L sodium hydroxide and 1 g/L sodium hydrosulfite, 10 min, 80 °C), and cleaned after being cooled to 50 °C, the polyester-dyed fabrics were reduced. The samples were rinsed in cold water and then dried by air.

### 2.3. Fastness Properties Tests

According to the tests of the American Association of Textile Chemists and Colorists [8], the fastness characteristics of the dyed samples were examined under various conditions, including rubbing, washing, perspiration by using a grey scale (grades 1–5) and light fastness by using a blue wool scale is used (grades 1–8).

### 2.4. Evaluation of Antibacterial Activities of the Despise Dyes

The disc agar diffusion method was used to investigate the antibacterial activity of the dispersed dyes **7a**–**f**. *Staphylococcus aureus*, *Bacillus subtilis*, *Escherichia coli*, and *Pseudomona aeruginosa* were selected as the four representative test microorganisms, together with yeast *Candida albicans* and the filamentous fungus *Aspergillus niger*. In the case of bacteria and yeast, nutrient agar plates were severely injected on a regular basis with 0.1 mL of 105–106 cells/mL. To assess the antifungal effects, 0.1 mL (10^6^ cells/mL) of the fungal inoculum was seeded onto Czapek-Dox agar plates. The inoculation plates were covered with dyes discs, which were then incubated at 30 °C for 48 h to promote the maximal growth of the organisms and 37 °C for 24 h for bacteria. By taking a millimetre (mm) measurement of the diameter of the zone of inhibition, the test agent’s antibacterial activity was ascertained. The experiment was run multiple times, and the average reading was recorded. Antibacterial and antifungal standards were neomycin (200 µg/mL) and cyclohexamide (200 µg/mL), respectively.

### 2.5. Color Strength

Colour intensity was measured using the Kubelka-Munk equation

$$K/S=\frac{{\left(1-R\;\right)}^{2}}{2R}-\frac{{\left(1-R^{0}\right)}^{2}}{2R^{0}}$$

K is the absorption coefficient, S is the scattering coefficient, and R is the decimal fraction of the reflectance of the dyed cloth, Ro is the decimal fraction of the reflectance of the non-dyed fabric.

### 2.6. Washing Fastness

To assess the washing fastness, the ISO 105-C02 method from 1989 was employed [10]. The test pieces are then immersed in an aqueous solution with a solution ratio of 5 g/L of non-ionic detergent (1:50) for 30 min at 60 °C with two samples of bleached cotton and wool fabric sandwiched between them. The samples were removed after a predetermined period of time, twice washed while intermittently applying hand pressure, and then dried. Assessments of washing fastness are made.

### 2.7. Rubbing Dfastness

The ISO 105-X12:1987 test technique was used to measure colour fastness to rubbing. The test is intended to determine how much colour might rub off of the surface of coloured materials onto another surface. Both dry and wet materials can be used in the current test.

#### 2.7.1. Wet Test

On the crockmeter’s base, the test sample was laid flat. There was a white test cloth mounted. The test specimen was placed on the covered finger, which was then made to glide back and forth 20 times. The white test sample was then taken out and evaluated using the staining on the grey scale.

#### 2.7.2. Dry Test

Water was applied to the white test sample in a thorough (65%) manner. As before, the process was followed. Prior to analysis, the white test samples were air dried.

### 2.8. Perspiration Fastness

The creation of two solutions—acid and alkaline—follows test method ISO 105-E041988. The procedure that is performed to check for resistance is as follows. A 5 cm 4 cm dyed patch was stitched between two white swatches to produce the composite design. Mixed samples were immersed in both solutions for 15 to 30 min while being forcefully agitated and squeezed to ensure complete soaking. A force of around 4–5 kg is used to hold the sample in place between two glass or plastic plates. After that, the combined sample plates were kept upright in a 37.2 °C oven for 4 h. Utilising the grey scale for colour change, the effect on the test sample’s colour is illustrated and assessed.

### 2.9. Light Fastness

Testing for light fastness in accordance with ISO 105-B02. In the test, a carbon arc lamp is employed, and it is run nonstop for 35 h. Finally, it can be deduced that the type of fabric into which the dye has been incorporated is what causes the colour of the dyed fabric to intensify with increasing dye concentration. This is because different fabrics contain different chemical groups, and these substituents can greatly influence the light fastness index of a dye on a particular fabric. The distribution of the incoming radiation’s wavelengths; not all absorptions equally start the bleaching process. The rate at which certain colourants fade can be considerably impacted by the humidity and chemistry of the environment. The hue variations of the examined materials were noted using the blue scale.

### 2.10. Evaluation of Cytotoxic Effects Synthesized Dyes

Human breast cancer cell line MCF-7; human hepatocellular carcinoma cell line HepG-2; colon cancer cell line HCT-116; and lung cancer cell line A-549 are examples of mammalian cell lines. the VACSERA Tissue Culture Unit provided. Chemicals: Trypan blue dye, dimethyl sulfoxide (DMSO), and crystal violet were all acquired from Sigma in St. Louis, MO, in the United States. Lonza was used to acquire foetal bovine serum, DMEM, RPMI-1640, HEPES buffer solution, L-glutamine, gentamycin, and 0.25% Trypsin-EDTA. When assessing cytotoxicity activities, the established methodology was used.

## 3. Results and Discussions

**Scheme 1:**
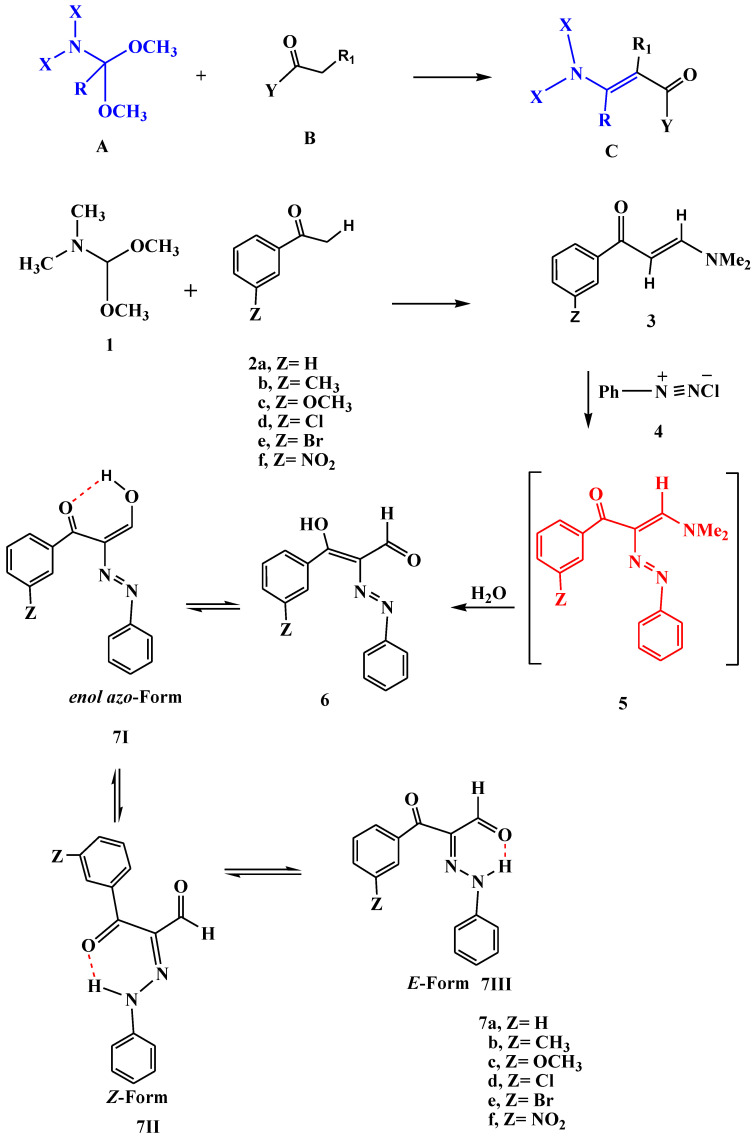
Synthesis of the novel ao disperse dyes **7a–f**.

3-(dimethylamino)-1-phenylprop-2-en-1-ones (C) could be readily synthesized by condensation reaction of dialkylamino dimethyl acetals (A) with methylketones (B). Through the condensing reaction of (DMFDMA) **1** with methylketones **2a–f** in xylene, 3-(dimethylamino)-1-phenylprop-2-en-1-ones **3a–f** were produced in respectable yields. We have previously accounted for the requirements for the efficient synthesis of 3-(dimethylamino)-1-phenylprop-2-en-1-ones from methylketones and DMFDMA, including microwave irradiation as a source of heating [19]. 3-oxo-3-phenyl-2-(2-phenylhydrazono)propanals disperse dyes **7a–f** were produced by coupling of 3-(dimethylamino)-1-phenylprop-2-en-1-ones **3a–f** with phenyl diazonium chloride **4** (Scheme 1).

When diazotized phenyl diazonium chloride **4** was coupled with enaminones derivatives **3a–f**, a product of coupling and dimethylamine hydrolysis was also eliminated. ^1^H NMR of this product show that it exists in DMSO as an equilibrium mixture of *enol azo*- form **7I**, *Z*-form **7II** and the *E*-form **7III**, as ^1^H NMR revealed that the major constituent in this equilibrium mixture was for the *E*-form, also these forms are stabilized by hydrogen bonding. This is completely consistent with what Al-Zaydi et al. [23] have published or what we have published before that compound **7III** (phenyl diazonium chloride was *p*-ClC_6_H_4_) exists in the *E*-form and we have published an X-ray crystalographic analysis of it [26].

### 3.1. Dye Uptake

Disperse dyes are the most significant class of dyes employed in the dying of polyester materials, and this fact is well known in the dye sciences and textile industries. By applying a high temperature of about 130 °C or dyeing at 100 °C with a carrier present, the dyeing rate can be increased to a commercially excellent and thus extremely acceptable standard. In order to increase dye absorption, speed up dyeing, and reduce dyeing temperature, dyeing polyester with carrier has been extensively explored [1,2,3]. We can state that the dye structure affects how the carrier affects the dyeing rate. Increasing the dyeing temperature of polyester fabrics to 130 °C results in a significant increase in the K/S values because the polyester structure is made more swellable and less compressible, which makes it easier for the chains to move partially and show improved dye absorption. Additionally, the high temperature aids in the disintegration of the dye molecules and their solubility, speeding up and enhancing the diffusion and penetrating power of the dye.

The information in Table 1 and Table 2 shows that almost all colored polyester fabrics transmitted a similar hue when the dye’s hue was given as (h*) values. Positive estimates of (b*) showed that dyed polyester fabric color hues moved in a yellowish direction. The colorimetric parameters values obtained for the high temperature dyeing polyesters were given in Table 1 and expressed as color strength (K/S), which shows that disperse dye **7a** has a good affinity towards polyester fabrics and has color strength K/S 16.55. The phenyl moiety of compound 6a was replaced with an electron-donating group to demonstrate the link between structure and activity. This substitution effectively provided a strong colour strength by producing the analogues **7b** and **7c** (K/S = 15.83 and 15.79, respectively) from C_6_H_4_CH_3_-*m*, or C_6_H_4_OCH_3_-*m*. However, when the phenyl moiety of compound **7a** was replaced with an electron-withdrawing group, such as (C_6_H_4_Br-*m*, or C_6_H_4_NO_2_-*m*), the resulting compounds **7e**, and **7f** had a weaker color (K/S = 15.62 and 15.51). With the exception of that, dye **7d** (C_6_H_4_Cl-*m*) gave satisfactory color strength K/S equals (16.32).

The colour strength was measured using the UltraScan Pro (Hunter Lab, Reston, VA, USA) 10° observer with D65 illuminant, d/2 viewing geometry, and a measurement area of 2 mm.

According to colorimetric analysis, by using high temperature dyeing process, the colour of polyester fabric dyed with disperse dye **7a** (the value of L = 83.37) was lighter and brighter than the colours of polyester fabrics dyed with disperse dyes **7a** and **7c** (the values of L = 77.30, and 78.48).

Similarly, the colorimetric parameters values obtained for the low temperature dyeing polyesters were given in Table 2 and expressed as color strength (K/S), which shows that disperse dye **7a** has a good affinity towards polyester fabrics and has color strength K/S 11.20. The phenyl moiety of compound **7a** was replaced with an electron-donating group to demonstrate the link between structure and activity. This substitution effectively provided a strong colour strength by producing the analogues **7b** and **7c** (K/S = 12.07 & 14.29, respectively) from C_6_H_4_CH_3_-*m*, or C_6_H_4_OCH_3_-*m*. However, when the phenyl moiety of compound **7a** was replaced with an electron-withdrawing group, such as (C_6_H_4_Br-*m*), the resulting compounds **7f** had a weaker color (K/S = 10.69). With the exception of that, dye **7d** (C_6_H_4_Cl-*m*) gave higher color strength K/S equals (16.06).

### 3.2. Fastness Properties

Polyester fabrics were dyed using the innovative six disperse dyes **7a**–**f**. The dyed polyester fabrics at 130 °C (Table 3) or at 100 °C (Table 4) displayed good results for rubbing, moderate results to light fastness and very good results for washing and perspiration fastness.

### 3.3. Antibacterial Activities of the Despise Dyes

**Figure 1:**
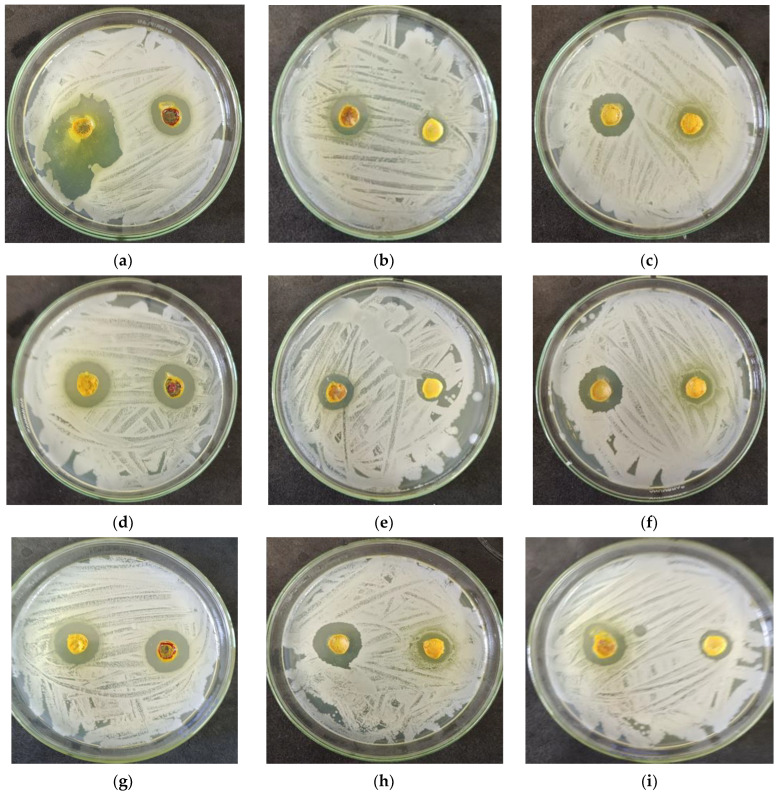
The photographs show the zone of inhibition of the disperse dyes: (**a**) disperse dyes **7a** & **7b** against *S. aureus*, (**b**) disperse dyes **7c** & **7d** against *S. aureus*,(**c**) disperse dyes **7e** & **7f** against *S. aureus*,(**d**) disperse dyes **7a** & **7b** against *B. subtilis*, (**e**) disperse dyes **7c** & **7d** against *B. subtilis*, (**f**) disperse dyes **7e** & **7f** against *B. subtilis*, (**g**) disperse dyes **7a** & **7b** against *P. aeruginosa*, (**h**) disperse dyes **7c** & **7d** against *P. aeruginosa*,(**i**) disperse dyes **7e** & **7f** against *P. aeruginosa*. The results showed that these dispersion dyes that we constructed have encouraging antibacterial activity, which gives them an added value.

From Table 5 and Figure 1 we find that in general all of the dyes under study have biological activity against at least four microbes. Dye **7a** has excellent biological activity for *Staphylococcus aureus*, *Pseudomonas aeruginosa*, *Candida albicans* and *Aspergillus niger*, very good activity for *Escherichia coli* and good activity for *Bacillus subtilis*. Dye **7b** has excellent biological activity for Pseudomonas aeruginosa and Aspergillus niger and very good activity for *Staphylococcus aureus*, *Bacillus subtilis* and *Escherichia coli* and good activity for *Candida albicans*.

Dye **7c** has very good biological activity for *Staphylococcus aureus*, *Bacillus subtilis*, *Pseudomonas aeruginosa*, *Candida albicans* and *Aspergillus niger* and has no activity for *Escherichia coli*. Dye **7d** has very good biological activity for *Staphylococcus aureus*, *Bacillus subtilis*, *Pseudomonas aeruginosa*, and *Candida albicans* and has no activity for *Escherichia coli* and *Aspergillus niger*.

Dye **7e** has excellent biological activity for numbers *Bacillus subtilis* and *Escherichia coli* and has very good activity for numbers *Staphylococcus aureus*, *Pseudomonas aeruginosa*, *Candida albicans* and *Aspergillus niger*. Dye **7f** has very good biological activity for *Staphylococcus aureus*, *Bacillus subtilis*, and *Pseudomonas aeruginosa*, and no activity for *Escherichia coli*, *Candida albicans*, and *Aspergillus niger*.

In general, dyes that contain electron-giving groups in their chemical structure have better biological activity than those dyes that contain electron-withdrawing groups in their chemical structure

### 3.4. In Vitro Cytotoxicity Screening

**Figure 2:**
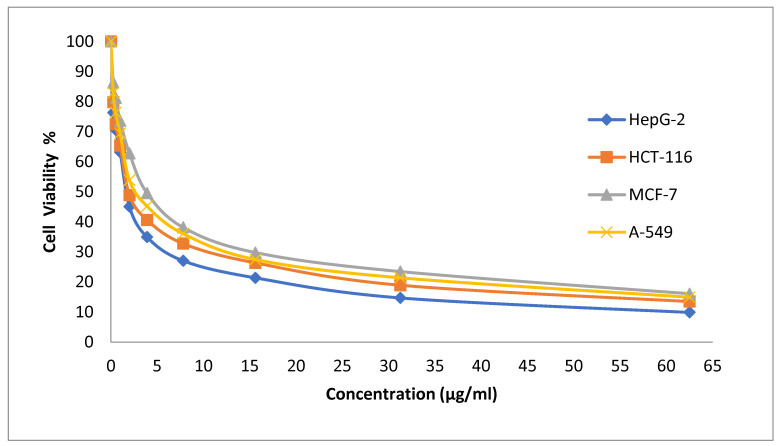
Anticancer activities of dye **7a**.

**Figure 3:**
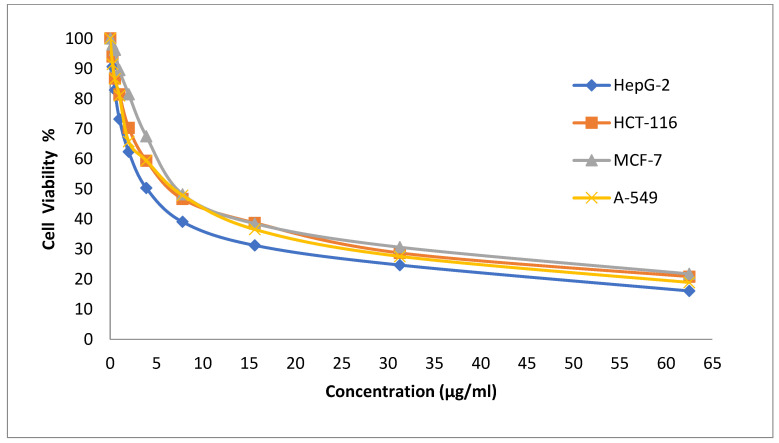
Anticancer activities of dye **7b**.

**Figure 4:**
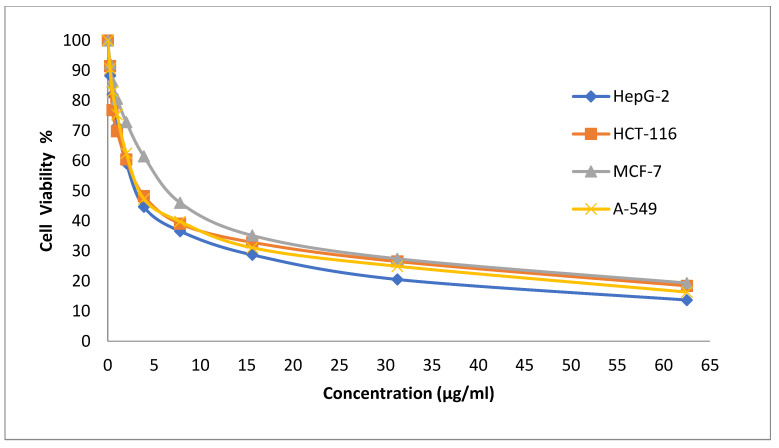
Anticancer activities of dye **7c**.

**Figure 5:**
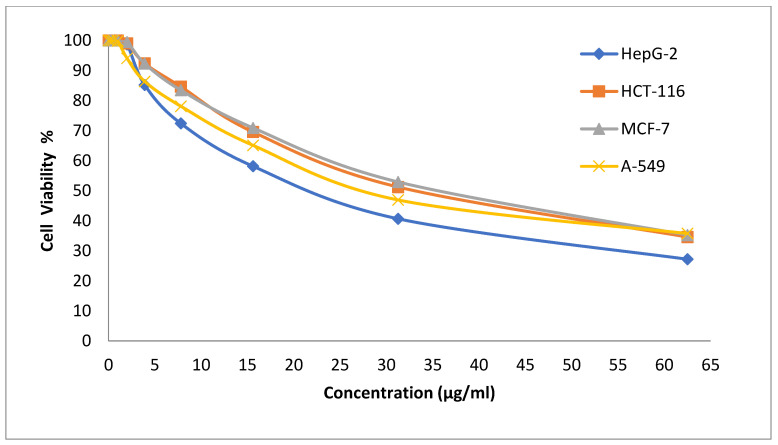
Anticancer activities of dye **7d**.

**Figure 6:**
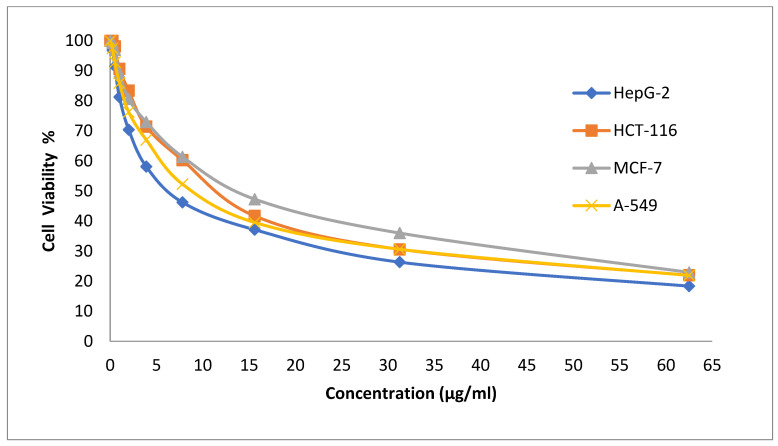
Anticancer activities of dye **7e**.

**Figure 7:**
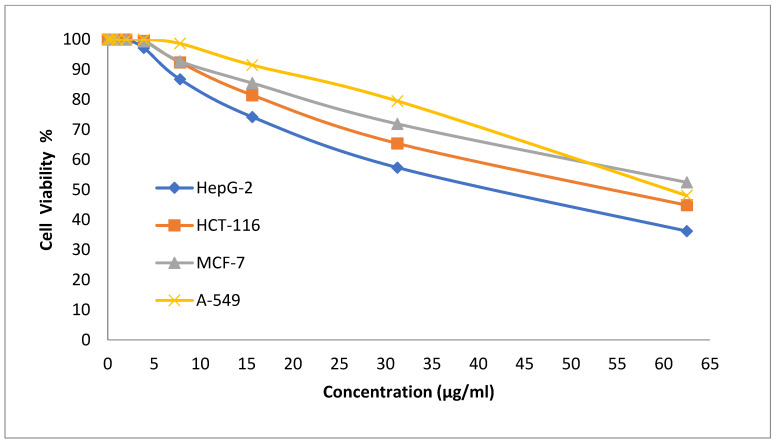
Anticancer activities of dye **7f**.

The four human cell lines HepG-2 (for hepatocellular carcinoma), HCT-116 (for colon carcinoma), MCF-7 (for breast cancer), and A-549 (for lung cancer) were used to test the anticancer properties of the newly synthesized dispersion dyes **7a–f**. Different concentrations of the six disperse dyes were used to calculate the IC_50_ (µg/mL) values (the concentration required to inhibit 50% of the growth of the culture when the cells are exposed to the tested disperse dyes for 48 h). In three separate assays, cytotoxic activity was measured as the mean IC_50_. When compared to cisplatin as a reference medication, the results are shown in Table 6 and Figure 2, Figure 3, Figure 4, Figure 5, Figure 6 and Figure 7, which demonstrate that compounds **7a** and **7c** have the maximum cytotoxic activity against the HePG-2, HCT-116, MCF-7, and A-549 cells. Additionally, compound **7a** was more effective than cisplatin in killing HePG-2, HCT-116, MCF-7, and A-549 cells, with IC_50_ values of 1.73, 1.93, 3.93, and 2.88 g/mL in each case, compared to 3.69, 2.54, 5.68, and 7.49 µg/mL.

Disperse dye **7a** has very significant activity, as shown by Table 6 and Figure 2, Figure 3, Figure 4, Figure 5, Figure 6 and Figure 7, with IC_50_ values of 1.73, 1.93, 3.93, and 2.88 µg/mL in HePG-2, HCT-116, MCF-7, and A-549 cells, respectively. By replacing the phenyl moiety of compound **7a** with electron-donating groups such as C_6_H_4_CH_3_-*m*, and C_6_H_4_OCH_3_-*m*, compound **7b** and compound **7c** were produced, with IC_50_ values of 3.99, 6.79, 7.45, 7.09 and 3.27, 3.71, 6.80, and.65 µg/mL, respectively.

This substitution effectively produced strong anticancer activity against the four cell lines with an IC_50_ that was comparable to cisplatin. Interestingly, compound **7c** was more effective than cisplatin at killing HePG-2 and A-549 cells, with IC_50_ values of 3.27 and 3.65 µg/mL in each case, compared to 3.69 and 7.49 µg/mL.

Additionally, compound **7f** had comparable cytotoxic effects to cisplatin against the HePG-2, HCT-116, MCF-7, and A-549 cells.

Contrarily, the anticancer activity of compounds **7d**, **7e**, and **7f** was diminished when the phenyl moiety of compound **7a** was replaced by an electron-withdrawing group such as (C_6_H_4_Cl-*m*, C_6_H_4_Br-*m*, or C_6_H_4_NO_2_-*m*) (IC_50_ = 22.85, 33.55, 36.39, 28.55 and 6.57, 12.12, 14.11, 9.22 and 42.12, 54.63, 69.74, 60. We concentrated our attention on the most promising compounds, **7b** and **7d**, as an intriguing starting point for the development of a new class of antimicrobial agents in light of the findings presented in this work and taking into account that this preliminary study produces conclusive evidence regarding a structure antimicrobial activity relationship. Compounds **7a** and **7c**, however, showed encouraging inhibitory effect against the four tumor cells that were examined. To expand the scope of these new heterocyclic frameworks’ potential as leads for the development of fresh chemotherapeutic drugs, we believe that research in this area should be promoted.

## 4. Conclusions

We obtained derivatives of 3-(dimethylamino)-1-phenylprop-2-en-1-ones with an easy and fast method without using a catalyst. This method gave excellent yields when methyl ketones were reacted with dimethylformamide dimethylacetal. 3-(dimethylamino)-1-phenylprop-2-en-1-ones have been reacted with phenyl diazonium chloride to provide novel disperse dyes. The new dyes were used in dyeing polyester fabrics that gave bright colors ranging from yellow to orange using low temperatures at 100 °C or high temperatures at 130 °C. The color strength that was obtained proved that dyeing polyester fabrics using a high temperature method is much better than dyeing using low temperature methods. We studied the different fastness properties of dyed fabrics, which gave good results for their fastness against washing, perspiration, and rubbing and moderate results for fastness to light. The possibilities of these new disperse dyes including their activity against some types of bacteria and different cancers were studied. Compounds **7a**, **7c** and **7b**, **7e** showed excellent and very good potential inhibitory effect against the four tumor cells that were tested (i.e., HePG-2, HCT-116, MCF-7, and A-549, according to in vitro cytotoxic activity tests). Moreover, we obtained promising results for the possibility of using these dyes as antibacterial and anticancer agents.

## Figures and Tables

**Table 1:** Color strengths of polyester fabrics dyed at 130 °C.

Dye No.	K/S	λ_max_	L*	a*	b*	C*	h*
**7a**	16.55	405	83.37	−9.72	74.00	74.64	97.49
**7b**	15.83	405	77.30	1.52	73.74	73.76	88.82
**7c**	15.79	405	78.48	−1.65	70.36	70.38	91.35
**7d**	16.32	405	81.87	−6.71	75.37	75.67	95.08
**7e**	15.62	405	78.38	1.13	71.56	71.57	89.09
**7f**	15.51	405	77.41	2.03	71.72	71.75	88.37

**Table 2:** Color strengths of polyester fabrics dyed at 100 °C.

Dye No.	K/S	λ_max_	L*	a*	b*	C*	h*
**7a**	11.20	405	77.94	1.51	58.27	58.29	88.52
**7b**	12.07	405	77.32	4.32	65.91	66.06	86.25
**7c**	14.29	405	77.34	0.04	63.80	63.80	89.96
**7d**	16.06	405	82.99	−7.66	69.52	69.94	96.29
**7e**	10.69	405	78.30	1.38	58.43	58.44	88.65
**7f**	12.06	405	80.48	−1.91	60.51	60.54	91.81

**Table 3:** Fastness properties of the polyester fabrics dyed at 130 °C.

Dye No.	Rubbing Fastness		Washing Fastness				Perspiration Fastness								Light Fastness
							Acid				Alkaline				
	Dry	Wet	St.	St. *	St **	Alt.	St.	St. *	St **	Alt.	St.	St. *	St **	Alt.	
**7a**	4	4	4	4	4	4	4	4	4	4	4–5	4–5	4–5	4	4
**7b**	4	4	4	4	4	4	4	4	4	4	4	4	4	4	3–4
**7c**	4	4	4	4	4	4	4	4	4	4	4–5	4	4	4	3
**7d**	4	4	4	4	4	4	4	4	4	4	4–5	3–4	4	4	4
**7e**	4–5	4–5	4	4	4	4	4	4	4	4	4	4	4–5	4	4
**7f**	3–4	3–4	4	4	4	4	4	4	4	4	4	4	4–5	4	3–4

Alt.: alteration; St.: staining on cotton; St. *: staining on wool and St **.: staining on polyester.

**Table 4:** Fastness properties of the polyester fabrics dyed at 100 °C.

Dye No.	Rubbing Fastness		Washing Fastness				Perspiration Fastness								Light Fastness
							Acid				Alkaline				
	Dry	Wet	St.	St. *	St **	Alt.	St.	St. *	St **	Alt.	St.	St. *	St **	Alt.	
**7a**	4	4	4	4	4	4	4	4	4	4	4–5	4–5	4–5	4	4
**7b**	3–4	3	4	4	4	4	4	4	4	4	4–5	4–5	4–5	4–5	4
**7c**	4	3–4	4	4	4	4	4	4	4	4	4–5	4–5	4–5	4	4
**7d**	4	4	4	4	4	4	4	4	4	4	4	4	4	4	4
**7e**	3–4	3–4	4	4	4	4	4	4	4	4	4	4	4–5	4	3–4
**7f**	3–4	3–4	4	4	4	4	4	4	4	4	4	4	4–5	4	4

Alt.: alteration; St.: staining on cotton; St. *: staining on wool and St **.: staining on polyester.

**Table 5:** The antimicrobial activity of dyes **6a–f** against different test microbes.

Dye No.	Clear Zone (mm)					
	*Staphylococcus aureus*	*Bacillus subtilis*	*Escherichia coli*	*Pseudomonas aeruginosa*	*Candida albicans*	*Aspergillus niger*
**7a**	19	8	16	19	20	21
**7b**	15	16	14	19	8	24
**7c**	13	15	0	17	16	14
**7d**	12	13	0	14	14	0
**7e**	16	19	14	18	17	12
**7f**	12	12	0	14	0	0

**Table 6:** Antitumor activities of the disperse dyes **7a–f**.

Dye No.	Cytotoxic Activity (IC_50_ µg/mL)			
	HepG-2	HCT-116	MCF-7	A-549
**7a**	1.73 ± 0.16	1.93 ± 0.34	3.93 ± 0.34	2.88 ± 0.13
**7b**	3.99 ± 0.49	6.79 ± 0.67	7.45 ± 0.54	7.09 ± 0.34
**7c**	3.27 ± 0.36	3.71 ± 0.52	6.80 ± 0.51	3.65 ± 0.15
**7d**	22.85 ± 0.87	33.55 ± 1.73	36.39 ± 3.43	28.55 ± 1.86
**7e**	6.57 ± 0.59	12.12 ± 0.83	14.11 ± 0.95	9.22 ± 0.47
**7f**	42.12 ± 1.82	54.63 ± 5.11	69.74 ± 4.06	60.54 ± 4.32
**Cisplatin**	3.69 ± 0.27	2.54 ± 0.23	5.68 ± 0.44	7.49 ± 0.65

## Data Availability

Not applicable.
